# Association of Voriconazole Trough Plasma Concentration with Efficacy and Incidence of Hepatotoxicity in Iranian Patients with Hematological Malignancies

**DOI:** 10.22037/ijpr.2020.112330.13688

**Published:** 2021

**Authors:** Hamidreza Taghvaye-masoumi, Molouk Hadjibabaie, Maryam Ghadimi, Morvarid Zarif-Yeganeh, Mohammad Vaezi, Ardeshir Ghavamzadeh

**Affiliations:** a *Department of Clinical Pharmacy, Faculty of Pharmacy, Guilan University of Medical Sciences, Rasht, Iran. *; b *Department of Clinical Pharmacy, Faculty of Pharmacy, Tehran University of Medical Sciences, Tehran, Iran. *; c *Research Center for Rational Use of Drugs, Tehran University of Medical Sciences, Tehran, Iran. *; d *Hematology-Oncology and Stem Cell Transplantation Research Center, Shariati Hospital, Tehran University of Medical Sciences, Tehran, Iran. *

**Keywords:** Voriconazole trough concentration, Invasive aspergillosis, Hematological malignancies, Hepatotoxicity, Efficacy

## Abstract

There are conflicting data regarding the association between plasma concentration of voriconazole (VCZ) and both efficacy and safety. This study investigates the association of VCZ trough plasma level with clinical efficacy and hepatotoxicity in the Iranian population suffering hematological malignancies. This cross-sectional study was performed on adult Iranian patients (age ≥ 18 years) with hematological malignancies undergoing treatment with oral or intravenous VCZ for proven or probable invasive *aspergillosis.* Plasma concentrations of VCZ were measured at two time points on day 4 and 14 during the study period. A total of 60 VCZ trough concentrations of 30 patients were drawn on days 4 and 14 after the initiation of treatment. There was no definite correlation between the mean plasma concentration of VCZ and VCZ dosage (*p = *0.134, r = 0.280). In multivariable model, only plasma concentration of VCZ on day 14 was associated with the incidence of hepatotoxicity (*p = *0.013; OR = 1.42, 95% CI = 1.07-3.24). Plasma trough concentration neither on day 4 nor on day 14 was related to the treatment response. No significant association was observed between the mean plasma concentration of VCZ and 3-month patients’ survival (*p = *0.696). To conclude, VCZ trough concentration may not be a predictor of treatment response or 3-month patients’ survival. However, the wide inter- and intra-patient variability of VCZ plasma concentration coupled with the observed association between VCZ trough level and the incidence of hepatotoxicity would pose the question regarding the potential benefit of VCZ concentration monitoring.

## Introduction

Invasive *aspergillosis *(IA) is a life-threatening infection that generally occurs in immunocompromised patients, including neutropenic individuals with hematological malignancies and hematopoietic stem cell transplantation (HSCT) recipients (1). Voriconazole (VCZ), the drug of choice for the treatment of *aspergillosis* infection, is being administered for the prevention and treatment of invasive fungal infections (IFI) in these populations (2, 3).

VCZ has non-linear pharmacokinetics with narrow therapeutic index due to the saturable metabolism in adults (4). According to the previous studies, VCZ therapeutic plasma reference range appears to be approximately 1.5 to 5.5 mcg/mL and supra-therapeutic concentration is associated with the incidence or severity of adverse events (5, 6). 

VCZ, like other triazole agents, has a favorable safety profile with most common adverse events including gastrointestinal (GI) symptoms, hepatotoxicity, visual disturbances, neurological toxicity, and rash. These adverse events are generally well tolerated; however, VCZ might cause hepatic failure that can impact patients’ outcome (7, 8). VCZ-induced hepatotoxicity might present as mild liver function abnormalities or even as severe hepatitis. There are contradictory findings regarding the association between VCZ trough levels and risk of hepatotoxicity (9, 10).

On the other hand, the sub-therapeutic concentration of VCZ might be associated with the lack of efficacy and poor outcome. Limited and inconsistent data from relatively small studies have assessed the association of VCZ plasma concentration and treatment response (11-14).

In the present study we aimed to investigate the association between VCZ trough plasma concentration with both efficacy and hepatotoxicity in Iranian patients suffering hematological malignancies or undergoing HSCT.

## Experimental

*Patients and drug administration*

This cross-sectional study was performed at the Hematology-Oncology Research Center and Stem Cell Transplantation (HORCSCT) of Shariati Hospital affiliated to Tehran University of Medical Sciences, Tehran, Iran from March 2015 to November 2016. The study protocol was approved by the Ethics Committee for Human Research at Tehran University of Medical Sciences (Ethics code: IR.TUMS.REC.1394.1100). All participants signed written informed consent forms.

Adult Iranian patients (age ≥ 18 years) with hematological malignancies undergoing treatment with oral or intravenous (IV) VCZ for proven or probable invasive *aspergillosis,* were enrolled in the study. Exclusion criteria included pregnancy, lactation, pre-existing liver dysfunction (Alanine transaminase (ALT), Aspartate aminotransferase (AST), Alkaline Phosphatase (ALP), Gamma-glutamyltransferase (GGT), and total bilirubin above the upper limit of normal) or kidney dysfunction (creatinine clearance less than 50 mL/min) before VCZ initiation. Upper limit of ALT, AST, ALP, GGT and total bilirubin were considered 35 unit/L, 35 unit/L, 308 unit/L, 50 unit/L and 1.2 mg/L, respectively. Patients who received VCZ for less than 14 days or did not cooperate during the follow up period were also excluded.

Proven or probable IA was defined according to the criteria established by the European Organization for Research and Treatment of Cancer and Mycoses Study Group of the National Institute of Allergy and Infectious Diseases. The *aspergillus* galactomannan test was judged to be positive if the measured value was ≥ 5.0 pg/mL. Halo sign, air-crescent sign, or cavities within area of consolidation were considered as fungal infection manifestation in radiographic findings (15).

Intravenous VCZ (Vfend ®, Pfizer, USA) was administered as a 6 mg/kg loading dose for two doses, followed by 200 mg twice daily infused over 1 h. When the patients could tolerate medication given by mouth, they were switched to oral VCZ (Vfend ®, Pfizer, USA) 200 mg twice daily one hour before or one hour after a meal. Due to insufficient drug availability and high cost of drug, maintenance dosing of intravenous VCZ were not weight based.

The patients’ baseline characteristics and information on VCZ dose and route of administration, length of hospitalization after VCZ initiation, and concomitant medications during VCZ therapy were recorded. Patients were followed for three months after the initiation of VCZ by a clinical pharmacy resident with two separate phone numbers from the patient and his/her family.

*Measurement of VCZ plasma concentration*

Venous blood samples were collected in heparinized tube on day 4 and 14 after initiation of VCZ; 15–30 min prior to the next dose. Day 4 was selected for the first sampling time because the drug has definitely reached to the steady state concentration at this time point. Day 14 was considered the second day of sampling, for most patients with *aspergillosis* infection admitted for at least 14 days in our center. The blood samples were centrifuged (3000 rpm, 4 °C, 10 min) and the plasma transferred to 2 mL polypropylene micro-tubes. The plasma samples were stored in -70 °C until assay. Plasma concentration of VCZ was quantified using a standardized High-Performance Liquid Chromatography (HPLC) assay with technique extracted from Khoschsorur *et al. *(16). Agilent 1260 infinity (USA) was used for HPLC with column of Ultra C18 5 µm (250 × 4.6 mm) Restek (USA). The measurable range of plasma concentration with this method was 0.1 to 20 mg/L with a correlation coefficient of 0.9989. VCZ was supplied by Pfizer® (USA) and ketoconazole (as internal standard) was supplied by Merck® (Germany). 

*Monitoring for hepatic dysfunction*

For evaluation of hepatic toxicity, liver enzyme tests from the day of VCZ initiation were recorded. Hepatic laboratory tests, including ALT, AST, ALP, GGT, and total bilirubin were recorded daily in a prespecified sheet throughout the first 14 days of VCZ therapy. Daily assessment of these parameters was performed as a protocol in this center for all patients. The severity of VCZ induced liver dysfunction was graded as described by the National Cancer Institute (NCI) of the National Institutes of Health (version 4.0: CTCAEv4) (17). Grades 3 and 4 were defined as severe hepatic dysfunction (Table 1). Naranjo Scale was used by 2 independent investigators to determine whether hepatic dysfunction is actually due to VCZ rather than other factors. Probability in Naranjo Scale was categorized as definite, probable, possible, or doubtful (18). Doubtful cases were not considered as VCZ induced adverse effect.

*Evaluation of efficacy*

The assessment of response to antifungal therapy was done based on the clinical (signs and symptoms of infection, including fever, manifestations of lung and brain involvement or disseminated *aspergillosis* and inflammatory markers such as ESR and CRP), radiological (CX-Ray or lung CT findings), and mycologic (galactomannan serum level) findings. The assay of serum *aspergillus* galactomannan antigen and chest X-ray or lung CT scan was performed at least weekly. Patients showed lack of improvement or worsening in at least two of the above three criteria after treatment, were considered non-responders to therapy (19). Fungus-related mortality was defined as death with continuing signs of fungal infection (20). 

*Statistical analysis*

Statistical analysis were performed using SPSS software (Statistical Package for the Social Sciences, version 21.0; SPSS Inc., Chicago, Illinois, USA). Sample size was estimated based on the treatment failure rate according to the results of Miyakis *et al.* (21). Kolmogorov-Smirnov test was conducted to assess the normality of distributions of continuous variables. Descriptive statistics were expressed as mean ± standard deviation or median (minimum-maximum), based on variables’ distributions, for quantitative variables and frequency (Percentage) for qualitative ones. Spearman rank test was used for evaluation of the correlation between VCZ dosage and plasma concentration of VCZ. For the analysis of hepatotoxicity and response to the treatment, univariate and multivariable logistic regression analyses were performed. The association between VCZ plasma concentration and 3-month survival was tested by Kruskal-Wallis test. Fisher’s exact test was performed to assess whether the 3-month survival related to the incidence of hepatotoxicity. *P*-values less than 0.05 were considered statistically significant. 

## Results

*Patient characteristics and voriconazole plasma concentrations*

Of 49 patients who were suspected to have probable or proven *aspergillosis *infection, a total of 30 patients were included in the study. All patients received VCZ for the treatment of probable *aspergillosis* for at least 14 days. Among them, 46.6% (n = 14) received both oral and intravenous VCZ while the remaining, 53.3% (n = 16) received only intravenous formulation. Acute myeloid leukemia was the most common underlying condition. The demographic and clinical characteristics of patients are summarized in Table 2.

**Figure 1:**
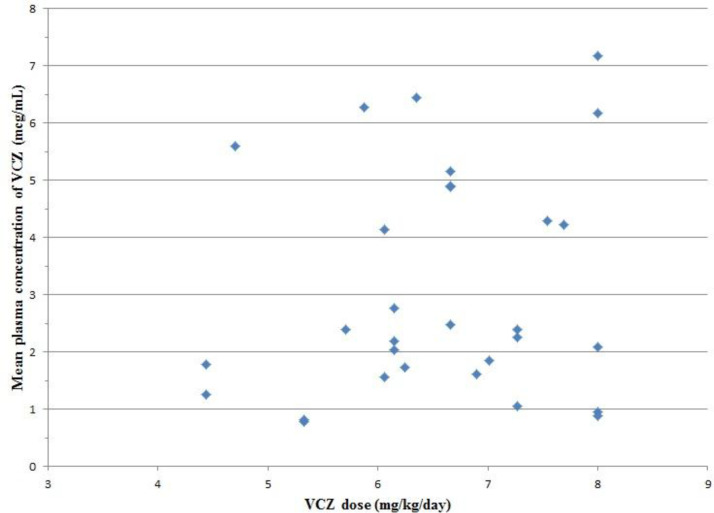
Correlation between mean plasma concentration of voriconazole and voriconazole dose (*p = *0.451, r = 0.143). VCZ: Voriconazole

A total of 60 VCZ trough concentrations of 30 patients were drawn on days 4 (C1) and 14 (C2) after the initiation of treatment. The median VCZ concentration was 2.47 mcg/mL (0.45-7.79 mcg/mL) in total measurements. The median VCZ concentration on days 4 and 14 were 2.45 mcg/mL (0.45-6.58 mcg/mL) and 2.5 mcg/mL (0.45-7.79 mcg/mL), respectively. Concentrations of < 1 mcg/mL and > 5.5 mcg/mL were detected in 15% (n = 9) and 16.6% (n = 10) of total measurements, respectively. The median maintenance dose of VCZ was 6.66 mg/kg/day (4.44-8 mg/kg/day). There was no definite correlation between the mean plasma concentration of VCZ and VCZ dosage (*p = *0.451, r = 0.143) (Figure 1). Neither C1 nor C2 correlated significantly with drug dosage (*p = *0.358, r = 0.174 and *p = *0.303, r = 0.195). 

The median of VCZ level changes between C1 and C2 was 32% (2.9-266.6%). Drug level on day 14 increased in 63.3% of patients and decreased in others. 

*Voriconazole plasma concentration and response to the treatment*

Forty percent (n = 12) of our patients did not respond to antifungal therapy after 14 days of treatment. The univariate logistic regression analysis demonstrated that age, gender, VCZ dosage, and VCZ plasma concentration on days 4 and 14 did not relate to response to the treatment on day 14 (Table 3). However, response to the treatment on day 14 was reversely associated with patients’ weight (*p = *0.044). Finally, the variables entered into a backward stepwise multivariable logistic regression model, and results showed that only weight was associated with response to the treatment on day 14 (*p = *0.044).

Forty three percent of patients were alive at the end of the observation period. There was no significant association between the mean plasma concentration of VCZ and 3-month patients’ survival (*p = *0.696). Neither C1 nor C2 was significantly related to 3-month patients’ survival (*p = *0.093, and *p = *0.268).

*Voriconazole plasma concentration and hepatotoxicity*

Among 30 patients who were followed for 14 days, 36.6% (n = 11) experienced hepatic enzyme abnormalities. Scores based on Naranjo causality approach for diagnosis of VCZ induced hepatotoxicity ranged from 3 to 7 (possible or probable) with an average of 5. Data about VCZ trough levels and their association with hepatotoxicity was shown in Table 4. The mean time to hepatotoxicity onset after VCZ initiation was 8.64 ± 2.80 days. Of the 11 patients with hepatic dysfunction, severe hepatic dysfunction was observed only in 1 patient with the mean VCZ concentration of 7.185 ± 0.605 mcg/mL. The pattern of hepatic dysfunction was hepatocellular (n = 7) or mixed (n = 4). The abnormal liver function tests were not observed in patients with mean VCZ concentration <1.62 mcg/mL. The univariate logistic regression analysis showed that gender, VCZ dosage, and VCZ plasma concentration on days 4 and 14 were associated with VCZ-induced hepatotoxicity (Table 5). Finally, the variables entered into a backward stepwise multivariable ordinal logistic regression model, and results revealed that only the VCZ plasma concentration on day 14 was significantly associated with the incidence of hepatotoxicity (*p = *0.013; OR = 1.42, 95% CI = 1.07-3.24). Additionally, 3-month patients’ survival was significantly associated with the incidence of hepatotoxicity (*p = *0.037). 

## Discussion

In the present study, we found high and unpredictable inter- and intra- patient variations in VCZ plasma concentrations among patients with hematological malignancies. This study showed that there is no significant association between trough plasma concentration of VCZ and clinical efficacy or 3-month patients’ survival. However, the incidence of VCZ-induced hepatotoxicity was significantly associated with VCZ concentration on day 14 of treatment. To our knowledge, this is the first published study (searched on PubMed-MEDLINE up to April 2017) which evaluates VCZ plasma level on two specified days of treatment in Iranian patients with probable or proven *aspergillosis*.

Intra-patient variability of trough plasma concentration up to 266.6% during the first 14 days of treatment demonstrated that multiple therapeutic drug monitoring (TDM) of VCZ is desirable after the initiation of therapy. It was found that the VCZ plasma concentrations drawn 4 days after the initiation of therapy might be higher (in 36.6% of participants) or lower (in 63.3% of participants) than the plasma concentrations measured on day 14. The causes of concentration variation between days 4 and 14 might be related to disease conditions or physiologic changes, including GI absorption, liver metabolism, or protein bindings (22). It appears that the severity of illness can not only impact the outcome, but may also reduce the elimination of VCZ (23). VCZ trough concentration ranged from 0.45 to 7.79 mcg/mL in our study which refers to high inter-patient variability of VCZ plasma concentration. *CYP2C19* genetic polymorphisms, possible drug-drug interactions, and differences in drug dosing (based on weight) are the major possible reasons for this inter-patient variability (22, 24).

 In consistent with Ueda *et al.* study, we found no significant correlation between VCZ dose and trough concentration on days 4 and 14. The lack of correlation is possibly contributed to *CYP2C19* genetic polymorphisms and drug-drug interaction, demonstrating that the trough level of VCZ cannot be predicted (19). 

Some potent *CYP3A4* inducers, such as phenytoin, rifabutin, rifampin, barbiturates, and efavirenz are known to interact with VCZ and can reduce VCZ plasma concentration (7). Our patients did not receive any of these medications. The trough level of VCZ in one case with underlying disease of acute lymphoblastic leukemia in both days 4 and 14 were ˂ 1 mcg/mL while the patient was receiving the correct maintenance dose (4 mg/kg twice daily). The notable concurrent medication for this patient was 8 mg of dexamethasone twice daily. It has been demonstrated that co-administration of glucocorticoids significantly increases VCZ elimination and reduces drug plasma level (25). 

In the present study, neither C1 nor C2 was related to the treatment response on day 14 or 3-month patients’ survival. There is conflicting data regarding the association between VCZ plasma concentration and treatment efficacy. Troke *et al.* identified a non-linear relationship between average plasma concentration and treatment efficacy. They found that patients with trough levels between 3 to 4 mcg/mL show maximum response rate (26). Further, a recent study demonstrated that VCZ concentrations did not relate to treatment response and suggested that routine monitoring of drug level had limited clinical role in patients suffering from hematological malignancy (12). However, growing body of evidence suggests potential links between treatment response and VCZ trough concentration. In this way, Pascual *et al. *in a cohort of 52 patients receiving VCZ for treatment of known or suspected IFI, demonstrated that trough level of VCZ is an important predictor of treatment efficacy and clinical outcome. They found that VCZ levels ≥ 1.5 mcg/mL are significantly associated with higher rates of treatment success (6). A multi-center study of 201 patients receiving VCZ revealed that the incidence of treatment failure was significantly greater in patients with trough levels < 1.7 mcg/mL (27). An association between the mean plasma concentration of VCZ and treatment response indicates a reasonable need for TDM. In our study, the lack of association between plasma concentration of VCZ and treatment response might be related to small sample size or diversities in baseline disease severity. Patients’ weight was the only variable affected treatment response and patients with lower weight had better clinical response compared with patients with higher weight. This might be because patients at lower weight received effective doses of VCZ.

 Considering the differences in the antifungal therapy patients received, we cannot definitely conclude that 3-month survival does not relate to plasma concentration of VCZ. For example, treatment schedules varied in some patients (including the addition of caspofungin and amphotericin B or withdrawal of VCZ) after the first 14 days of therapy. It should be noted that we did not draw trough levels after the first 14 days. Therefore, due to the unpredictable inter-patient variability of VCZ concentration in prolonged antifungal therapy, the trough level may not be as high as it was in the first 14 days of treatment. On the other hand, various factors such as hepatotoxicity, the severity of underlying disease, and other concomitant infections could impact the patients’ survival (22, 23). 

The incidence range of liver enzyme abnormalities following treatment with VCZ was between 6.3 to 51% in different studies (9). In the present study, liver enzyme abnormalities were observed in 36.6% of patients during the first 14 days of treatment. We observed a significant association between the incidence of liver enzyme abnormality and VCZ plasma concentrations on days 4 and 14. However, in multivariable model, only the plasma concentration on day 14 was related to VCZ-induced hepatotoxicity. Previous studies demonstrated that VCZ trough concentration < 4 mcg/mL is desirable for the prevention of hepatotoxicity. Y. Suzuki *et al.* observed that sustained high trough level of VCZ is associated with higher incidence of hepatotoxicity and suggested that VCZ trough levels should be maintained below 4 mcg/mL (28). Pascual’s study failed to demonstrate a significant correlation between plasma concentration of VCZ and hepatotoxicity. However, the incidence of hepatotoxicity increases in patients with trough levels ≥ 1.4 mcg/mL (6). Our results are consistent with findings of previous studies; the liver abnormality rate was significantly higher at mean VCZ concentrations of >4 mcg/L (72.7%) as compared to concentrations of ≤ 4 mcg/ml (15.79%). Given that the mean time to hepatotoxicity onset after VCZ initiation was 8.64 ± 2.80 days and only the VCZ plasma concentration on day 14 was related to hepatotoxicity, monitoring trough plasma concentration of VCZ during the first week of treatment would be a rational approach to prevent VCZ-induced hepatotoxicity. 

In addition to the small sample size, this study has several other limitations. First, all of the patients had a probable *aspergillosis* diagnosis and no patients were considered to have definite (proven) infection. Hence, the possible false positive diagnosis might mislead our assessment. Second, the patients received different treatment schedules after the first 14 days. Third, we did not measure plasma concentrations of VCZ after the first 14 days. Fourth, *CYP2C19* genetic polymorphism, as the main elimination metabolic pathway of VCZ, was not assessed.

**Table 1:** Hepatic dysfunction grades as described by NCI (17).

**Feature**	**Grade 0**	**Grade 1** ^*^	**Grade 2** ^*^	**Grade 3** ^*^	**Grade 4** ^*^
ALT	Normal	>1.0-2.5	>2.5-5.0	>5.0-20	>20
AST	Normal	>1.0-2.5	>2.5-5.0	>5.0-20	>20
Alkaline Phosphatase	Normal	>1.0-2.5	>2.5-5.0	>5.0-20	>20
GGT	Normal	>1.0-2.5	>2.5-5.0	>5.0-20	>20
Total bilirubin	Normal	>1.0-1.5	>1.5-3.0	>3.0-10	>10

The values expressed the multiples of the upper limit of the normal range (ULN).

ALT: Alanine transaminase; AST: Aspartate aminotransferase; GGT: Gamma-glutamyltransferase; NCI: National Cancer Institute.

**Table 2:** Demographic and clinical characteristics of patients (n = 30).

**Characteristics**	**Values**
**Sex**	
Male, n (%)	16 (53.3)
Female, n (%)	14 (46.7)
**Age (years)**	
Median (minimum-maximum)	37 (18-55)
**Weight (kg)**	
Median (minimum-maximum)	60 (50-85)
**Underlying disease**	
AML, n (%)	15 (50)
Post-HSCT, n (%)^ ¶^	12 (40)
NHL, n (%)	2 (6.7)
ALL, n (%)	1 (3.3)
**Route of administration**	
IV, n (%)	14 (46.7)
Switch from IV to oral therapy, n (%)	16 (53.3)
**Voriconazole** **dose (mg/kg/day)**	
Mean (± SD)	6.55 ± 1.05
**Voriconazole** **concentration (mcg/mL)**	
Median (minimum-maximum)	2.47 (0.45-7.79)

ALL: Acute lymphoblastic leukemia; AML: Acute myeloid leukemia; IV: Intravenous; HSCT: Hematopoietic stem cell transplantation; NHL: Non-Hodgkin lymphoma. ^¶^Patients hospitalized after HSCT on days +30 or thereafter for treatment of transplantation complications.

**Table 3:** Association between selected variables and response to the treatment on day 14

**Variables**	**Odds ratio**	***p*** **-value**	**(95% CI)**
Age (year)	1.00	0.971	0.94-1.06
Sex	2.2	0.299	0.49-9.74
Weight (kg)	0.90	0.044	0.82-0.99
Voriconazole dosage (mg/kg/day)	2.24	0.062	0.96-5.24
Voriconazole plasma concentration on day 4 (mcg/mL)	1.35	0.181	0.86-2.13
Voriconazole plasma concentration on day 14 (mcg/mL)	1.29	0.214	0.86-1.94

**Table 4:** Association between trough plasma concentrations of voriconazole and the incidence of hepatotoxicity

**Voriconazole** **concentration (mcg/mL)**	**Voriconazole** **induced hepatotoxicity, n (%)**	**Voriconazole** **induced severe hepatotoxicity, n (%)**
< 2	9 (1/11)	0 (0/11)
2 - 4	25 (2/8)	0 (0/8)
> 4	72.7 (8/11)	9 (1/11)

**Table 5:** Association between selected variables and voriconazole-induced hepatotoxicity

**Variables**	**Odds ratio**	***p*** **-value**	**(95% CI)**
Age(year)	0.95	0.109	0.89-1.01
Sex	0.19	0.046	0.04-0.96
Weight (kg)	0.81	0.004	0.70-0.93
Voriconazole dosage (mg/kg/day)	5.50	0.003	1.76-17.15
Voriconazole plasma concentration on day 4 (mcg/mL)	2.98	0.001	1.59-5.57
Voriconazole plasma concentration on day 14 (mcg/mL)	2.64	<0.0001	1.54-4.52

## Conclusion

In conclusion, among Iranian patients with hematological malignancy, VCZ trough concentration may not be a predictor of treatment response or 3-month patients’ survival. However, the wide inter- and intra-patient variability of VCZ plasma concentration coupled with the observed association between VCZ trough level and the incidence of hepatotoxicity is posing the question regarding the potential benefit of VCZ concentration monitoring. 
